# SlowMo, a digital therapy targeting reasoning in paranoia, versus treatment as usual in the treatment of people who fear harm from others: study protocol for a randomised controlled trial

**DOI:** 10.1186/s13063-017-2242-7

**Published:** 2017-11-02

**Authors:** Philippa A. Garety, Thomas Ward, Daniel Freeman, David Fowler, Richard Emsley, Graham Dunn, Elizabeth Kuipers, Paul Bebbington, Helen Waller, Kathryn Greenwood, Mar Rus-Calafell, Alison McGourty, Amy Hardy

**Affiliations:** 1 Department of Psychology, King’s College London, Institute of Psychiatry, Psychology and Neuroscience, P077 Henry Wellcome Building, De Crespigny Park, London, SE5 8AF UK; 2 South London and Maudsley NHS Foundation Trust, London, UK; 3 Department of Psychiatry, Oxford University, Oxford, UK; 4 School of Psychology, University of Sussex, Brighton, UK; 5 Sussex Partnership NHS Foundation Trust, Worthing, UK; 6 Centre for Biostatistics, School of Health Sciences, The University of Manchester, Manchester Academic Health Science Centre, Manchester, UK; 7 Division of Psychiatry, University College London, London, UK; 8 Oxford Health NHS Foundation Trust, Oxford, UK

**Keywords:** Delusions, Persecutory, Fast and slow thinking, Belief flexibility, Jumping to conclusions, mHealth, eHealth, Digital therapy, User-centred design

## Abstract

**Background:**

Paranoia is one of the most common symptoms of schizophrenia-spectrum disorders, and is associated with significant distress and disruption to the person’s life. Developing more effective and accessible psychological interventions for paranoia is a clinical priority. Our research team has approached this challenge in two main ways: firstly, by adopting an *interventionist causal approach* to increase effectiveness and secondly, by incorporating *user-centred inclusive design methods* to enhance accessibility and usability. Our resultant new digital intervention, SlowMo, intensively targets a reasoning style associated with paranoia, *fast thinking,* characterised by jumping to conclusions and belief inflexibility. It consists of an easy-to-use, enjoyable and memorable digital interface. An interactive web-based app facilitates delivery of face-to-face meetings which is then synchronised with an innovative mobile app for use in daily life.

**Methods/Design:**

We aim to test the clinical efficacy of SlowMo over 24 weeks to determine the mechanisms through which it reduces paranoia, and to identify participant characteristics that moderate its effectiveness. In a parallel-group randomised controlled trial, with 1:1 allocation, 360 participants with distressing persecutory beliefs will be independently randomised to receive either the SlowMo intervention added to treatment as usual (TAU) or TAU, using randomly varying permuted blocks, stratified by paranoia severity and site. Research workers will be blind to therapy allocation. The primary outcome is paranoia severity over 24 weeks; our hypothesised mechanism of change is reasoning; moderators include negative symptoms and working memory; and secondary outcomes include wellbeing, quality of life, and service use. The accessibility, usability and acceptability of the digital platform will be assessed.

**Discussion:**

SlowMo has been developed as the first blended digital therapy to target fears of harm from others through an inclusive design approach. In addition to testing its efficacy, this trial will add to our understanding of psychological mechanisms in paranoia. The study will examine the usability and adherence of a novel digital therapy, including an app for self-management, in a large sample of people affected by severe mental health difficulties.

**Trial registration:**

ISRCTN registry, ID: ISRCTN32448671. Registered prospectively on 30 January 2017. Date assigned 2 February 2017.

**Electronic supplementary material:**

The online version of this article (doi:10.1186/s13063-017-2242-7) contains supplementary material, which is available to authorized users.

## Background

People often experience distressing fears about other people intentionally causing them harm: this is known as paranoia [1]. The severity of paranoia lies on a continuum, ranging from fleeting ideas that someone on the street might be laughing at us, to more elaborate and persistent beliefs (sometimes called persecutory delusions) such as that the secret services are trying to have us killed. Paranoia is one of the most common symptoms of schizophrenia-spectrum disorders, and is associated with significant distress and disruption to the person’s life [2]. This results in increased use of services, including inpatient admissions, and high costs to mental health care providers. Developing effective interventions for paranoia is, therefore, a clinical priority. The National Institute for Health and Care Excellence (NICE; 2014) recommends cognitive-behavioural therapy for psychosis (CBTp), including paranoia [3]. However, there are significant challenges to access, engagement, adherence and effectiveness [4–6]. CBTp has relatively high training and delivery costs, which limits availability. Even when it is offered, people may be reluctant to engage in therapy, and can struggle to remember what is discussed or apply new learning to daily life. Recent meta-analytical studies of CBTp have found small- to medium-sized beneficial effects on paranoia, and a pressing need to improve outcomes has been identified [6]. Our research team has approached this challenge in two main ways: firstly by adopting an *interventionist causal approach* [7] to increase CBTp effectiveness and secondly by incorporating *user-centred inclusive design methods* to enhance accessibility and usability. Our resultant new digital intervention, SlowMo, aims to improve the appeal, ease of use, memorability and clinical effectiveness of psychological therapy for people who fear harm from others.

The interventionist causal approach to improving therapy effectiveness involves first identifying mechanisms that play a causal role in paranoia (e.g. reasoning, worry, negative self-beliefs, safety behaviours and sleep dysfunction) and then developing tailored interventions to target these causal processes [8, 9]. These targeted interventions are anticipated to reduce paranoia severity through different pathways given the multifactorial causality of paranoia. For example, a recent randomised controlled trial of a brief intervention focussed on worry processes demonstrated that reductions in this mechanism accounted for improvements in paranoia [10]. In contrast, SlowMo works by intensively targeting a certain type of thinking associated with paranoia, which can be thought of as *fast thinking* [11–13]. Fast thinking is characterised by focussing on too little information (‘jumping to conclusions’) and belief inflexibility (high conviction in thoughts and a lack of consideration of alternative ideas [13]). It has been robustly linked to paranoia [8, 12–17]. Systematic attempts to modify this style of reasoning include group-based, metacognitive training (MCT) and, more recently, individual training (MCT+) developed by Moritz and colleagues [18]. Whilst earlier findings for MCT were promising, more robust designs have not shown consistent improvements in reasoning, or paranoia at long-term follow-up, particularly for those with more severe difficulties [19–21].

The SlowMo intervention builds on the important work of Moritz and colleagues and is the endpoint of a decade of development and testing in four studies targeting fast thinking in paranoia [12, 22–24]. The first versions of the intervention were tested iteratively, in three randomised studies and one case series, with developments over time in intervention content, duration and name, whilst always targeting aspects of fast thinking and paranoia [12, 22–24]. We found reductions in unhelpful fast thinking and improvements in paranoia severity. In an experimental study [12] designed to examine mediation, we found preliminary evidence that changes in belief flexibility mediated improvements in paranoia. Most recently, in a feasibility randomised controlled trial, 31 participants with paranoia were recruited and randomly allocated 2:1 to the Thinking Well intervention or treatment as usual (TAU) [24]. The intervention involved face-to-face sessions with a therapist, initially working on a brief computer-based programme targeting reasoning biases [23] followed by four additional therapy sessions (with no additional digital component) aimed at generalising the learning to real-world situations. We found reductions in fast thinking (belief flexibility and jumping to conclusions) and promisingly large effects (effect size *d* = 1.1) on paranoia severity. However, assessments were not blinded and the sample size was small. Further, whilst the acceptability of the intervention was high, participants suggested ways in which the intervention could be made more personalised, enjoyable and applicable to daily life. Our experimental work also indicated that people with more working memory difficulties and negative symptoms benefitted less from the therapy. This current iteration (SlowMo) shares the focus on reasoning of its predecessors (Maudsley Review Training Programme [23] and Thinking Well [24]). However, whilst previous versions of the intervention relied on verbal presentation of material using PowerPoint and pen-and-paper tools, SlowMo uses a website and app for interactive, multimodal communication of information, together with consistent use of normalising (everyday) language.

Incorporating digital technologies into psychological interventions presents unique opportunities for improving outcomes, understanding mechanisms of change and reducing costs [25–27]. However, to deliver meaningful change in health care, digital solutions need to be tailored to meet the specific needs of their users and to be trustworthy with regard to safety, privacy and effectiveness [28–31]. To meet this challenge, the development of SlowMo therapy has involved a *user-centred inclusive design* approach. Inclusive design aims to address the needs of the broadest range of users possible, a crucial issue given the heterogeneity of psychosis. Our design approach was informed by the Design Council’s double diamond method [32], which comprises the following phases: discover, define, develop and deliver. Importantly, stakeholders (service users, clinicians, researchers, technologists, innovation design engineers), including sampling of ‘extreme’ users [33], were involved from the outset, with iterative exploration, prototype testing and feedback informing the design and development of SlowMo.

SlowMo aims to assist people with paranoia by supporting them to notice their upsetting concerns and fast-thinking habits, and then providing them with strategies to *slow down for a moment* in order to focus on new information and develop safer thoughts [34]. It consists of an easy-to-use, enjoyable and memorable digital interface. Thoughts are visualised as bubbles, with different speeds, sizes and colours reflecting different thinking habits, intensities of emotion and coping tips. This simple visual metaphor aims to help people to understand that thoughts are transient, and that we can modify them by using coping strategies. Based on session content from earlier work, an interactive web-based app facilitates the delivery of face-to-face meetings. This is then synchronised with an innovative, mobile app for use in daily life, which is ‘native’ (i.e. one that runs on the phone and can work offline). Feasibility testing of an early prototype has been conducted with acceptability, usability and enjoyment assessed through a self-report 10-item User Experience Survey, adapted from use in a previous study examining the feasibility of a mobile app for the management of psychosis [35]. The measure generates a mean percentage for each dimension of user experience, ranging from 0 (totally disagree) to 100 (totally agree). Results were extremely positive, with high rates of acceptability, usability and enjoyment (>75%). Participants indicated that they significantly preferred the digital interface to conventional therapy materials. This is particularly encouraging in the context of digital interventions given recent evidence that overall uptake of a therapeutic app for psychosis delivered in a naturalistic setting was low [30]. Given these data on the digital interface, together with the proof-of-concept, feasibility and acceptability evidence from our four preliminary studies [12, 22–24], we are now well placed to test the SlowMo intervention in a fully powered, larger, methodically rigorous, multisite randomised controlled trial.

### Aims

We aim to test the clinical efficacy of SlowMo over 24 weeks compared to TAU to determine the mechanisms through which it reduces paranoia, and to identify participant characteristics that moderate its effectiveness (either by moderating the degree of change in the mechanism, or by influencing adherence to the intervention). We will test the hypothesis that changes in fast thinking mediate changes in our primary outcome of paranoia severity. Consistent with our interventionist causal approach, we do not hypothesise that worry is a mediator, as it is not targeted in the SlowMo intervention, even though changes in worry did mediate changes in paranoia in a recent trial, when it was the treatment target [10]. However, we will examine any observed effects. In addition, we have preliminary evidence of modifiers of treatment effects that we will also take the opportunity to investigate. Using a randomised controlled trial design, we have selected TAU as the comparator condition. This is because there is a very low penetration of evidence-based psychological treatment in the NHS, and thus the key efficacy question to address at this stage is whether SlowMo confers benefits over and above standard care. An important secondary goal is to evaluate mechanisms of action; the trial hypotheses concern reasoning, and are best tested where the control condition is inactive with respect to the targeted psychological processes.

The main research questions are as follows:

1. Is SlowMo efficacious in reducing paranoia severity over 24 weeks, when added to TAU, in comparison to TAU alone?
2. Does SlowMo reduce paranoia severity by improving fast thinking (reducing belief inflexibility and jumping to conclusions)?
3. Do participant characteristics (i.e. their cognitive capacities, specifically working memory and thinking habits; and their symptoms, specifically negative symptoms) moderate the effects of the intervention?
4. Does outcome differ by adherence to the intervention and is adherence predicted by the participants’ beliefs about their illness and about the intervention?
5. Does the SlowMo digital therapy platform have acceptable rates of usability, acceptability and adherence?
6. Does SlowMo lead to changes in the following secondary outcomes: other delusional symptoms, wellbeing, quality of life, self and others schemas, service use and worry

### Hypotheses

Primary hypotheses:

1. The intervention will reduce paranoia severity over 24 weeks
2. Fast thinking (belief inflexibility and jumping to conclusions) will improve in response to the intervention
3. Reductions in fast thinking will mediate positive change in paranoia severity

Secondary hypotheses:

4. Poorer working memory and more severe negative symptoms will negatively moderate treatment effects
5. Therapy adherence will moderate the effects of treatment on outcome and adherence will be predicted by beliefs about mental health problems
6. Worry will not mediate reductions in paranoia severity

## Methods/design

### Trial design

The study design is a parallel-group randomised controlled trial, with 1:1 allocation. Participants with distressing persecutory beliefs who meet the inclusion criteria (see below) will be independently randomised to receive either the SlowMo intervention added to TAU, or TAU. Independent randomisation (centrally administered independently of the trial team by the King’s Clinical Trials Unit (CTU)) will use an online system generating randomly varying permuted blocks, stratified by site and baseline paranoia severity. Stratification by paranoia severity will use a median split of ≥ 62 (Green Paranoid Thoughts Scale (GPTS) part B [36] based on data from [10]). Research workers will be blind to therapy allocation, to facilitate completion of unbiased and objective assessments. Adherence to the blindness procedure will be supported by the research coordinator and therapists having responsibility for the randomisation process and informing participants of randomisation outcome. Further, the blinding procedure will be explained to participants and they will be reminded not to inform research workers of therapy allocation. Breaks in blinding will be monitored and recorded. Embedded within the design will be measures to elucidate how the treatment works. For reporting the trial, the CONSORT (Consolidated Standards of Reporting Trials; http://www.consort-statement.org/) Statement will be followed, with consideration of the mHealth evidence reporting and assessment (mERA) [37] and CONSORT-EHEALTH Checklists [38]. For the protocol, the SPIRIT (Standard Protocol Items: Recommendations for Interventional Trials [39]) Checklist and Figure are provided in this paper see: Additional file 1.

### Participants

The inclusion criteria are as follows: aged 18 years and over; persistent (3 + months) distressing paranoia (as assessed using the Schedules for Clinical Assessment in Neuropsychiatry (SCAN, [40]) and scoring > 29 on the GPTS, part B, persecutory subscale [36]; diagnosis of schizophrenia-spectrum psychosis (F20-29, ICD-10 [41]); capacity to provide informed consent; sufficient grasp of English to participate in informed consent process, assessments and interventions.

Criteria for exclusion are as follows: profound visual and/or hearing impairment; inability to engage in the assessment procedure; currently in receipt of other psychological therapy for paranoia; primary diagnosis of substance abuse disorder, personality disorder, organic syndrome or learning disability.

Mobile ownership is not a criterion for participation, as android smartphones with the SlowMo mobile app will be provided.

Participants will be recruited from mental health services across three main trial sites in England with the same procedures followed at each site: South London and Maudsley NHS Foundation Trust, Sussex Partnership NHS Foundation Trust and Oxford Health NHS Foundation Trust. Up to six additional Patient Identification Centres, comprising NHS trusts geographically near to the main recruitment trust sites, will be used as required.

### Trial flowchart

**Fig. 1:**
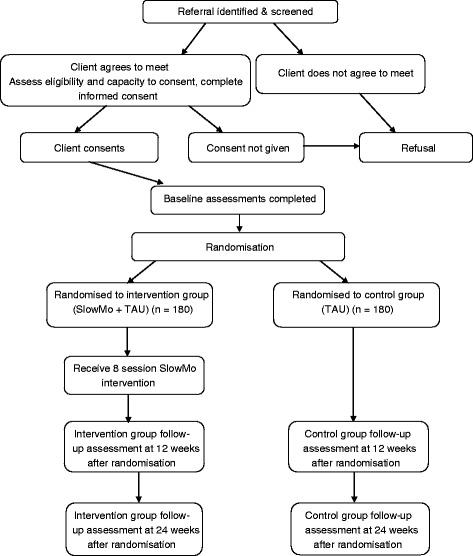
SlowMo trial design and recruitment flowchart

Figure 1 illustrates the trial/recruitment flowchart.

### Planned trial interventions

**Fig. 2:**
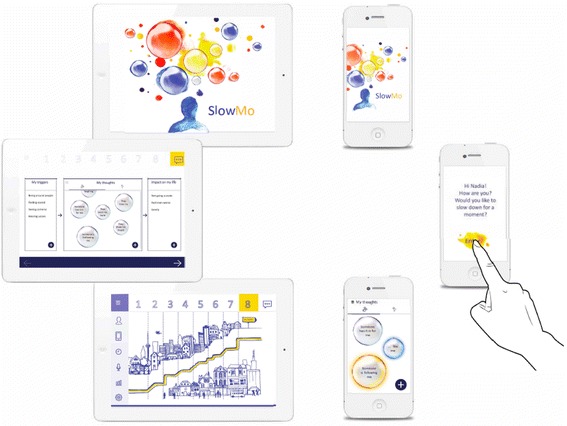
An overview of the SlowMo user interface (left: in-session digital platform; right: SlowMo app)

SlowMo therapy consists of eight individual, face-to-face sessions, of 60–90 min, delivered by trained therapists within a 12-week timeframe, assisted by a web-based app hosted on a touchscreen laptop, with interactive personal accounts and tasks. Initial sessions involve building the meta-cognitive skill of noticing thoughts and thinking habits (visualised as bubbles spinning faster or more slowly). People learn that everyone thinks fast at times, and this can be useful. However, thinking slowly can be helpful in dealing with stress and fears about other people. This key principle frames the sessions in which people are supported to try out tips to slow down for a moment, e.g. by considering the impact of mood and past experiences on concerns and by looking for safer alternative explanations. There is an emphasis throughout the intervention on practising the skills inside and outside sessions. Participants build confidence in managing paranoia, feeling safer in their daily life and working towards a valued goal. The overall session structure is fixed, but individual content is personalised throughout as participants record their individual worries, ways of feeling safer, key learning from each session, and a message for the week ahead. All of the personalised session content is synchronised with a native mobile app installed on a standard android smartphone to assist therapy generalisation into daily life. This allows people to notice their fears and thinking habits, and supports them to slow down for a moment, by providing strategies, encouraging them to audio- or text-record helpful new information and to generate safer thoughts. Recorded information is stored in a format whereby, when experiencing recurrent concerns, people can readily access what was previously useful. Optional notifications are available if people wish the app to check-in with them. The app is specifically designed for offline use, to minimise concerns about privacy and security. Participants are not given standardised instructions about when to use the app, rather the emphasis is on tailoring usage according to what is most helpful for the individual. Use of the app is monitored objectively through data input and system analytics. Please see Fig. 2 for an overview of the main SlowMo screens.

The development work has been done by Evolyst Ltd., a user-centred and evidence-based health care software development company. The design and development of the app has been informed by the British Standards Institute quality criteria and code of practice for health care apps (BSI; [42]). SlowMo uses a proprietary software platform developed using an Azure-based WCF (Windows Communication Foundation) Web Service, acting as an Application Programming Interface (API) to a Model View Controller (MVC) Asp.Net Web application; and a Xamarin.Android-based mobile application, allowing for use of the full Microsoft Stack and negating interoperability issues. SlowMo has currently been developed as a standalone product, given the lack of consensus on operating systems across the NHS trusts, and current interoperability issues.

TAU is care delivered to both randomised groups, according to national and local service protocols and best practice guidelines (specifically, NICE guidance on community mental health treatment for people with psychosis and the standards of community care required by the national regulators). Participation will not alter usual treatment decisions about medication and additional psychosocial interventions which remain the responsibility of the clinical team. A modified version of the Client Service Receipt Inventory [43] will be used to measure service use. Antipsychotic medication data will be extracted from medical records and dosages converted into chlorpromazine equivalents.

### Assessments and follow-up

#### Assessment of efficacy

**Fig. 3:**
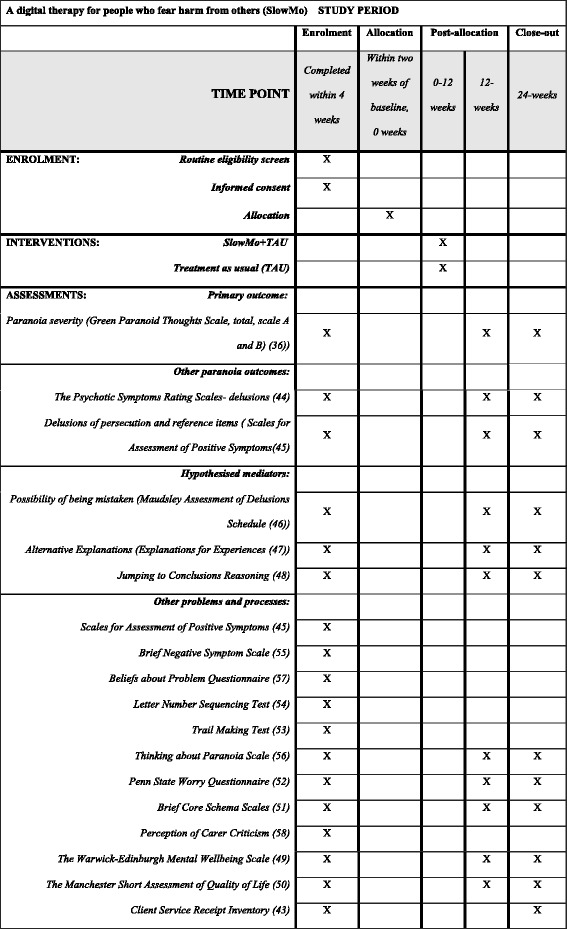
Standard Protocol Items: Recommendations for Interventional Trial (SPIRIT) Figure. A digital therapy for people who fear harm from others (SlowMo): schedule of enrolment, interventions and assessments

Participants will complete a range of self-report and interview-based measures to assess the impact of the interventions on primary and secondary outcomes, the hypothesised mediators, and other key processes implicated in paranoia and response to therapy. Assessments will be completed at baseline, 12 and 24 weeks. Every effort will be made to ensure that data collection and completeness is optimised throughout the trial, and to minimise attrition/loss to follow-up. Please refer to Fig. 3 (SPIRIT Figure) for details of assessment at each visit. Assessments will be audio-taped (after first establishing consent) to allow evaluation of adherence to the research protocol and assessment ratings.

The primary outcome is paranoia severity measured by the GPTS [36] over 24 weeks. The GPTS comprises two scales assessing thinking relevant to paranoia: ideas of social reference and persecution, rated over the preceding month. Each item is scored on a five-point Likert scale from 1 (‘not at all’) to 5 (‘totally’). A total score can be calculated ranging from 32 to 160, with higher scores reflecting higher levels of paranoia. Two 16-item subscales assess ideas of social reference (part A) and persecution (part B) relevant to paranoia.

Other paranoia outcomes:

1. The Psychotic Symptom Rating Scales-Delusions (PSYRATS-Delusions; [44]), consisting of six items which assess the following dimensions of delusions: amount of preoccupation with delusions, duration of preoccupation with delusions, conviction, amount of distress, intensity of distress and disruption to life caused by beliefs
2. The persecutory delusions and ideas of reference items from the Scales for Assessment of Positive Symptoms (SAPS; [45]), a semi-structured interview designed to assess the positive symptoms of psychosis

Hypothesised mediators are measured by changes in fast thinking assessed by:

1. Possibility of Being Mistaken (taken from the Maudsley Assessment of Delusions Schedule (MADS; [46]); Alternative Explanations from the Explanations of Experiences interview [47]). These are commonly used published methods of assessing lack of belief flexibility relating to delusional beliefs
2. Jumping to Conclusions (JTC) Beads Data-gathering Task [48] versions 85:15 and 60:40.

Please refer to Fig. 3 for details of secondary outcomes and other key processes hypothesised as moderators; these include published and established measures of wellbeing [49], quality of life [50], self and other schemas [51], service use [43], worry [52], cognitive tests [45, 53–55], other paranoia measures [56] and measures of beliefs about mental health problems and perceived relationship with carers [57, 58].

### Safety and adverse event assessment and monitoring and stopping rules

The occurrence of adverse events (AEs) will be monitored actively and systematically, following SPIRIT guidance for reporting of harms. AEs include: deaths; self-harm; serious violent incidents; complaints about therapy; and referrals to crisis care or admission to psychiatric hospital during therapy. A standard method of reporting will be employed, categorising events by severity (five grades, A–E). Subject to the approval by the independent chairperson of the Data Monitoring and Ethics Committee (DMEC, see below, ‘Research governance’), investigators will also determine whether an event is temporally related to the intervention, and whether it is unexpected or unexplained given the participant’s clinical course, previous conditions and history, and concomitant treatments. Following [59], the event will then be rated within five categories from ‘not related’ to ‘related’. Any associations between AEs and the SlowMo hardware or software will also be recorded. At each meeting of the DMEC, or at any time at the request of the DMEC chairperson, a full report of AEs will be reviewed. The DMEC will be responsible for investigating further, if there are concerns about unexpectedly high rates of AEs. This may involve the DMEC members being unblinded to the trial condition or seeking further data on AEs. If there are any ethical or safety reasons why the trial should be prematurely ended, they will advise the Trial Steering Committee (TSC) accordingly.

Individual participants will have the right to withdraw from the trial at any time. In addition the therapist, in collaboration with the participant and relevant clinical team, may decide to stop the therapy if it is directly associated with a worsening of mental state. Reasons for withdrawal from the study will be recorded. For the final reports of the trial, the numbers, types and severity of AEs by trial condition, as well as discontinuations, will be reported, using descriptive statistics (since there are no pre-specified hypotheses concerning AEs or harms, and, given the expected low frequency of AEs, the data will not be suitable for an intention-to-treat (ITT) statistical analysis).

The trial may be prematurely discontinued by the sponsor or chief investigator on the basis of new safety information or for other reasons given by the DMEC, the TSC, the regulatory authority or the Ethics Committee concerned. The trial may also be prematurely discontinued due to lack of recruitment or upon advice from the TSC, which will advise on whether to continue or discontinue the study and make a recommendation to the sponsor. If the study is prematurely discontinued, active participants will be informed and no further participant data will be collected.

### Accessibility, usability and acceptability assessments

Given the novelty of the digital therapy platform, its accessibility, usability and acceptability will be assessed in the SlowMo arm. This will be done through assessment of current mobile use and confidence at the beginning of therapy, monitoring of connectivity for the web app, system analytics data on the use of the platform, the User Experience Survey (adapted from [35]), and a service-user led qualitative interview with a sub-sample of those receiving SlowMo (*n* = 20).

### Therapy adherence assessments

In the SlowMo arm, therapy adherence will be assessed from the number and duration of sessions attended, and system analytics data on mobile app use. Therapy delivery will be evaluated in terms of fidelity to the treatment manual.

### Data management and security

All data will be anonymised at source. All personal data will be kept in a locked filing cabinet in a locked office and will be kept separate from all the research data. Therapy files will be kept in a secure office in the clinic and will not be accessible to the staff collecting the research outcome data. Data will be entered on a computerised database, held centrally and managed by King’s College London CTU, by research assistants using a secure network connection. Audio-recording equipment will be used to record assessments to check fidelity to assessment protocols and to ensure interrater reliability. The therapy sessions will be audio-recorded (with participant consent) for monitoring the intervention in terms of fidelity and competence. These audio files, named with a unique participant identifier, will be stored as computer files on secure NHS/university servers.

Security and privacy of information stored on the app has been considered throughout its development. If informed consent is provided, app data will only be synched during therapy sessions, over secure connections and stored on a password-protected, secure database. Data transferred will only contain a name (chosen by the person) and a Unique Device Identifier (UDID) which is generated automatically by the therapy platform, and will match the anonymised participant number. Participants can also opt to use the app in a fully offline mode. Participants will have the opportunity, if they wish, to password protect the handset with a pin number or password. During the informed consent process potential participants will be made fully aware of the data collected by the platform, and how data will be stored and used. Access to this privacy and security information is also available from the settings menu of the app, which consenting participants can access at any time.

### Data quality

Data quality will be ensured by close monitoring and routine auditing for accuracy throughout the data collection period. In order to ensure the accuracy of the data entered into the database, the main outcome measure entry will be checked for every participant by comparing the paper record with that on the database. An error rate of no more than 5% is acceptable. This will be done once all possible assessments for each time point have been completed. If the error rate is higher than 5%, advice will be sought from the trial statistician and methodologist regarding further data checking.

### Sample size

Recruitment of 360 participants will be split equally across sites. We have powered the study conservatively to detect a clinically meaningful 10-point reduction in the primary outcome measure (GTPS [36]); based on a standard deviation of 25, this is a 0.4 effect size [10]. We account for: clustering in the SlowMo arm with an intraclass correlation coefficient (ICC) = 0.01 with 10 therapists (no clustering in the TAU arm), 1:1 allocation, 0.05 significance level. Calculations used Clsampsi in Stata. A simple two-tailed *t* test with 150 people per group gives 90% power to detect an effect size of 0.4, and 80% for 0.35. In practice, power will be increased by using multiple regression. To allow for conservatively high 20% attrition we will recruit 360 patients at baseline split equally across three sites (120 per site, 60 per arm per site). For the mediational analyses, a sample of *N* = 300 has > 80% power to detect a proportion mediated of 40%, and > 70% power to detect a proportion mediated of 30%, corresponding to findings in our pilot work [12] (calculated using PowerMediation in R).

### Statistical analysis

We will report all participant flow, and analyses will be conducted on the ITT population: all participants will be randomised regardless of non-compliance with protocol or withdrawal from the study. Analyses will post-date final follow-up assessments, with due consideration of potential biases from loss to follow-up. The primary analysis will test for a treatment effect on the primary and secondary clinical outcomes. Random effects regression models allowing for clustering by both participants and therapists will be fitted to the repeated measures, controlling for treatment site, baseline paranoia severity and the corresponding baseline assessment for the outcome under investigation. We will allow for missing outcome data under the Missing At Random assumption [60]; we may also use inverse probability weighting to adjust for non-adherence to allocated treatment and other intermediate outcomes as predictors of future loss to follow-up [61]. Secondary analyses will test treatment-effect mechanisms, moderation and process/adherence effects using modern causal inference methods [62, 63]. The trial outcomes will comprise two parallel series of longitudinal data: one for the putative mediators (M) and one for the clinical outcomes (Y).

For the mechanistic analysis, to test for a treatment effect on the putative mediator, we will replace the clinical outcome with the mechanistic variable as the dependent variable in the random-effect models. If we separately demonstrate a treatment effect on both the putative mediator and on the clinical outcome, we will evaluate mediation in these parallel longitudinal data sets through the use of parallel growth curve and latent change models [64, 65]. These models preserve the basic mediation model by replacing observed variables with latent constructs – the growth factors driving the temporal responses, M_1_ to M_p_ and Y_1_ to Y_p_. Importantly, the mediational structure only applies to the slope growth or change factors since randomised treatments are independent of the intercept growth factors (baseline values). Growth curve and latent change models can be estimated by maximum likelihood and other methods using the software package Mplus [66]. The application of these methods to mechanism evaluation within EME (Efficacy and Mechanism Evaluation) trials is illustrated in [62]

The aim of these analyses is to demonstrate that the effect of treatment on the growth (change) in the clinical outcome (Y) is explained (caused) by its effect on the growth (change) in the mediator. The major challenge to a valid inference is that there may be confounding of the mediator and outcome. We will begin by allowing for baseline values of the mediator and of the clinical outcome, as in the analyses of the successful EME Worry Intervention Trial [10]. We will then check the sensitivity of the results to the possibility of hidden confounding (unmeasured variables) through the use of instrumental variable methods [62, 63].

### Research governance and patient and public involvement (PPI)

King’s College London is the research sponsor and the South London and Maudsley NHS Foundations Trust is co-sponsor. The trial has received a favourable ethical opinion from Camberwell St. Giles Research Ethics Committee (REC) (REC Reference: 16/LO/1862; IRAS: 206680). Any changes to the study protocol will be submitted to the REC and then communicated to all relevant parties (including the DMEC, TSC and study funders). The trial will be conducted in compliance with the principles of the Declaration of Helsinki [67], the Medical Research Council Guidelines for Good Clinical Practice [68] and in accordance with all applicable regulatory requirements including but not limited to the Research Governance Framework and the Mental Capacity Act 2005 [69]. The chief investigator (CI) will have overall responsibility for the trial data set and will permit trial-related monitoring, audits and REC review by providing the sponsor(s), and REC direct access to source data and other documents as required. A dedicated trial coordinator post will assist in the day-to-day management of the project reporting to the CI. A Trial Management Committee (TMC) will meet monthly: its membership will include the investigators and the trial coordinator and site coordinators. It will be chaired by the CI and will manage the day-to-day running of the study and oversee the preparation of reports to the TSC and DMEC. The TSC will meet at least annually and will include in its membership a lay member and access to consultation with a patient and public involvement (PPI) advisory group. The TSC’s purpose is to provide independent overall supervision of the trial, approving the protocol and amendments, and monitoring progress, through audits of recruitment and data completion rates and adherence to the protocol. It will provide independent advice on all aspects of the trial. A DMEC will be convened and will meet at least annually and report to the TSC. It will have access to all trial data and will receive regular reports on AEs. Membership of the DMEC will be fully independent of the trial team and will comprise two independent clinician researchers, one of whom will act as chair, and a statistician who will be independent of the applicants and of the TSC. The DMEC chair will be notified of any serious AEs as they occur, and with the DMEC will consider whether any interim analyses are warranted, review data and advise the TSC on any ethical or safety reasons why the trial should be prematurely ended. The PPI Advisory Group will advise on and contribute to recruitment, qualitative data collection and dissemination activities throughout the trial.

## Discussion

SlowMo has been developed as the first blended digital therapy to target fears of harm from others through an inclusive design approach. Improving the effectiveness and accessibility of psychological treatments for paranoia is a clinical health priority [6]. The current trial aims to achieve this in two ways. Firstly, adopting an interventionist causal treatment approach, SlowMo therapy tackles *fast thinking*, which research has shown to play a key role in the development and maintenance of distressing paranoia. Secondly, incorporating digital technologies into psychological interventions presents unique opportunities for developing effective and accessible treatments. However, the adoption of digital technology cannot of itself guarantee effective therapy. We therefore used an inclusive design approach in the development of SlowMo therapy, with stakeholder involvement at each stage and a clear focus on addressing the needs of the broadest possible range of users, including sampling of ‘extreme’ users [33].

Given the strong evidence for targeting reasoning as a treatment for paranoia [13, 70], our encouraging pilot data, and its inclusive user-centred design, SlowMo is expected to be highly acceptable and to lead to clinically worthwhile improvements in paranoia severity, working by supporting people to ‘slow down for a moment’ and reduce their reliance on fast thinking. The data from this study will also add significantly to our understanding of psychological mechanisms and change processes in paranoia. As well as providing valuable information for treatment development, evidence of mechanisms of action will inform the theoretical understanding of paranoia in a way that may itself shape future therapeutic initiatives. The trial will provide data on whether characteristics of participants (including working memory and negative symptoms) moderate the effects of the intervention on fast thinking, and also the effect on outcome of an adequate dose of treatment and therapy adherence. From the perspective of digital health, we will examine the usability and adherence of a novel digital therapy, including an app for self-management in daily life in a large sample of people affected by severe mental health difficulties. Uniquely, the mobile app allows for monitoring of fast and slow thinking in real time and is, therefore, well placed to advance our understanding of its role in paranoia [71].

In summary, the SlowMo trial has the potential to inform future stratified medicine approaches, the development of more targeted therapies and the applicability of digital health innovations with this population. The trial is funded for 31 months and began in February 2017. Final outcome assessments will be completed by summer 2019, and outcome results will become available in 2020. They will then be written up by the trial team and published in peer-reviewed journals. Participants will receive a summary of the results, and we will also disseminate findings more broadly through public engagement activities.

## Trial status

Recruitment of participants commenced in May 2017 and will be open until spring 2019. The date of first enrolment is May 2017.

**Key contacts:**

Professor Philippa Garety (CI) and Dr. Thomas Ward can be contacted for scientific queries.

Dr. Thomas Ward (trial coordinator) is the main contact for general trial-related queries.

**Chief investigator: Professor Philippa Garety**

PO Box 77, Henry Wellcome Building, The Institute of Psychiatry, Psychology and Neuroscience, King’s College London, De Crespigny Park, London SE5 8AF, UK

Tel: +44 (0)20 7848 5046; Fax: +44 (0)20 7848 5006

Email: philippa.garety@kcl.ac.uk

**Trial coordinator: Dr. Thomas Ward**:

Psychology, PO77, HWB, King’s College London, Institute of Psychiatry, Psychology and Neuroscience, De Crespigny Park, London SE5 8AF, UK

Tel: +44 (0) 207 848 0594; Fax: +44 (0) 207 848 5006

Email: thomas.ward@kcl.ac.uk

## Additional file

Additional file 1: SPIRIT 2013 Checklist: recommended items to address in a clinical trial protocol and related documents. (DOC 122 kb)

## Abbreviations

AE: Adverse event
API: Application Programming Interface
BCSS: Brief Core Schema Scales
BSI: British Standards Institute
CBTp: Cognitive-behavioural therapy for psychosis
CONSORT: Consolidated Standards of Reporting Trials
CTU: Clinical Trials Unit
DMEC: Data Monitoring and Ethics Committee
EME: Efficacy and Mechanism Evaluation
GPTS: Green Paranoid Thoughts Scale
ICC: Intraclass correlation coefficient
ICD: *International Classification of Diseases*
IRAS: Integrated Research Application System
ITT: Intention- to-treat
JTC: Jumping to Conclusions
KCL: King’s College London
MADS: Maudsley Assessment of Delusions Schedule
MANSA: Manchester Short Assessment of Quality of Life
MCT: Metacognitive training
MVC: Model View Controller
NICE: National Institute of Health and Clinical Excellence
NIHR: National Institute for Health Research
PPI: Patient and public involvement
PSYRATS: Psychotic Symptoms Rating Scales
R&D: Research and Development
RCT: Randomised controlled trial
REC: Research Ethics Committee
SAPS: Scale for the Assessment of Positive Symptoms
SLaM: South London and Maudsley NHS Foundation Trust
TAU: Treatment as usual
TMC: Trial Management Committee
TSC: Trial Steering Committee
UDID: Unique Device Identifier
WCF: Windows Communication Foundation

## References

[1] Freeman D, Garety PA, Bebbington PE, Smith B, Rollinson R, Fowler D (2005). Psychological investigation of the structure of paranoia in a non-clinical population. Brit J Psychiat.

[2] The Schizophrenia Commission. The abandoned illness: a report from the Schizophrenia Commission. London: Rethink Mental Illness; 2012.

[3] National Institute for Health and Care Excellence (NICE). Psychosis and schizophrenia: treatment and management. (Clinical guideline 178.) 2014. http://guidance.nice.org.uk/CG178. Accessed 30 Oct 2017. 31869041

[4] Haddock G, Eisner E, Boone C, Davies G, Coogan C, Barrowclough C (2014). An investigation of the implementation of NICE-recommended CBT interventions for people with schizophrenia. J Ment Health.

[5] Freeman D, Dunn G, Garety P, Weinman J, Kuipers E, Fowler D (2013). Patients’ beliefs about the causes, persistence and control of psychotic experiences predict take-up of effective cognitive behaviour therapy for psychosis. Psychol Med.

[6] van der Gaag M, Valmaggia LR, Smit F (2014). The effects of individually tailored formulation-based cognitive behavioural therapy in auditory hallucinations and delusions: a meta-analysis. Schizophr Res.

[7] Kendler KS, Campbell J (2009). Interventionist causal models in psychiatry: repositioning the mind-body problem. Psychol Med.

[8] Garety PA, Freeman D (2013). The past and future of delusions research: from the inexplicable to the treatable. Brit J Psychiat.

[9] Freeman D (2016). Persecutory delusions: a cognitive perspective on understanding and treatment. Lancet Psychiat.

[10] Freeman D, Dunn G, Startup H, Pugh K, Cordwell J, Mander H (2015). Effects of cognitive behaviour therapy for worry on persecutory delusions in patients with psychosis (WIT): a parallel, single-blind, randomised controlled trial with a mediation analysis. Lancet Psychiat.

[11] Kahneman D (2011). Thinking, fast and slow.

[12] Garety P, Waller H, Emsley R, Jolley S, Kuipers E, Bebbington P (2015). Cognitive mechanisms of change in delusions: an experimental investigation targeting reasoning to effect change in paranoia. Schizophrenia Bull.

[13] Ward T, Garety PA. Fast and slow thinking in distressing delusions: A review of the literature and implications for targeted therapy, Schizophr. Res. 2017. http://dx.doi.org/10.1016/j.schres.2017.08.04. 10.1016/j.schres.2017.08.045 PMC6336980 28927863

[14] Dudley R, Taylor P, Wickham S, Hutton P (2016). Psychosis, delusions and the ‘Jumping to Conclusions’ reasoning bias: a systematic review and meta-analysis. Schizophr Bull.

[15] McLean BF, Mattiske JK, Balzan RP (2016). Association of the jumping to conclusions and evidence integration biases with delusions in psychosis: a detailed meta-analysis. Schizophrenia Bull.

[16] So SH, Siu NY, Wong HL, Chan W, Garety PA (2016). ‘Jumping to conclusions’ data-gathering bias in psychosis and other psychiatric disorders—Two meta-analyses of comparisons between patients and healthy individuals. Clin Psychol Rev.

[17] So SH, Freeman D, Dunn G, Kapur S, Kuipers E, Bebbington P (2012). Jumping to conclusions, a lack of belief flexibility and delusional conviction in psychosis: a longitudinal investigation of the structure, frequency, and relatedness of reasoning biases. J Abnorm Psychol.

[18] Moritz S, Andreou C, Schneider BC, Wittekind CE, Menon M, Balzan RP (2014). Sowing the seeds of doubt: a narrative review on metacognitive training in schizophrenia. Clin Psychol Rev.

[19] Andreou C, Wittekind CE, Fieker M, Heitz U, Veckenstedt R, Bohn F (2017). Individualized metacognitive therapy for delusions: a randomized controlled rater-blind study. J Behav Ther Exp Psychiatry.

[20] van Oosterhout B, Krabbendam L, de Boer K, Ferwerda J, van der Helm M, Stant AD (2014). Metacognitive group training for schizophrenia spectrum patients with delusions: a randomized controlled trial. Psychol Med.

[21] van Oosterhout B, Smit F, Krabbendam L, Castelein S, Staring ABP, van der Gaag M (2016). Metacognitive training for schizophrenia spectrum patients: a meta-analysis on outcome studies. Psychol Med.

[22] Ross K, Freeman D, Dunn G, Garety P (2011). A randomized experimental investigation of reasoning training for people with delusions. Schizophrenia Bull.

[23] Waller H, Freeman D, Jolley S, Dunn G, Garety P (2011). Targeting reasoning biases in delusions: a pilot study of the Maudsley Review Training Programme for individuals with persistent, high conviction delusions. J Behav Ther Exp Psychiatry.

[24] Waller H, Emsley R, Freeman D, Bebbington P, Dunn G, Fowler D (2015). Thinking Well: a randomised controlled feasibility study of a new CBT therapy targeting reasoning biases in people with distressing persecutory delusional beliefs. J Behav Ther Exp Psychiatry.

[25] Moller AC, Merchant G, Conroy DE, West R, Hekler E, Kugler KC (2017). Applying and advancing behavior change theories and techniques in the context of a digital health revolution: proposals for more effectively realizing untapped potential. J Behav Med.

[26] Hollis C, Morriss R, Martin J, Amani S, Cotton R, Denis M (2015). Technological innovations in mental healthcare: harnessing the digital revolution. Brit J Psychiat.

[27] NHS England. NHS Five Year Forward View. 2014. Available from: https://www.england.nhs.uk/five-year-forward-view. Accessed 30 Oct 2017.

[28] Patel MS, Asch DA, Volpp KG (2015). Wearable devices as facilitators, not drivers, of health behavior change. JAMA.

[29] Singh K, Drouin K, Newmark LP, Lee J, Faxvaag A, Rozenblum R (2016). Many mobile health apps target high-need, high-cost populations, but gaps remain. Health Affair.

[30] Torous J, Roberts LW (2017). Needed innovation in digital health and smartphone applications for mental health: transparency and trust. JAMA Psychiat.

[31] Killikelly C, He Z, Reeder C, Wykes T. Improving adherence to online and mobile technologies for people with psychosis: a systematic review of new potential predictors of adherence. J Med Intern Res. 2017 (accepted manuscript). 10.2196/mhealth.7088 PMC5544896 28729235

[32] Design Council. Eleven lessons: managing design in eleven global brands. A study of the design process 2005. Available from: http://www.designcouncil.org.uk/sites/default/files/asset/document/ElevenLessons_Design_Council%20(2).pdf. Accessed 30 Oct 2017.

[33] Clarkson PJ, Coleman R, Keates S, Lebbon C (2003). Inclusive design—design for the whole population.

[34] Garety PA, Hardy A (2017). The clinical relevance of appraisals of psychotic experiences. World Psychiatry.

[35] Ben-Zeev D, Brenner CJ, Begale M, Duffecy J, Mohr DC, Mueser KT (2014). Feasibility, acceptability, and preliminary efficacy of a smartphone intervention for schizophrenia. Schizophrenia Bull.

[36] Green CE, Freeman D, Kuipers E, Bebbington P, Fowler D, Dunn G (2008). Measuring ideas of persecution and social reference: the Green et al. Paranoid Thought Scales (GPTS). Psychol Med.

[37] Agarwal S, LeFevre AE, Lee J, L’Engle K, Mehl G, Sinha C, et al. Guidelines for reporting of health interventions using mobile phones: mobile health (mHealth) evidence reporting and assessment (mERA) checklist. BMJ. 2016;352:i1174. 10.1136/bmj.i1174 26988021

[38] Eysenbach G, Grp C-E. CONSORT-EHEALTH: improving and standardizing evaluation reports of web-based and mobile health interventions. J Med Internet Res. 2011;13(4):e126. 10.2196/jmir.1923 PMC3278112 22209829

[39] Chan AW, Tetzlaff JM, Altman DG, Laupacis A, Gotzsche PC, Krleza-Jeric K (2013). SPIRIT 2013 Statement: defining standard protocol items for clinical trials. Ann Intern Med.

[40] World Health Organization (1992). SCAN Schedules for Clinical Assessment in Neuropsychiatry, Version 1.0.

[41] World Health Organization (2010). The ICD-10 classification of mental and behavioural disorders: diagnostic criteria for research.

[42] British Standards Institute. PAS 277: Health and wellness apps—Quality criteria across the life cycle—Code of practice. London: London, British Standards Institute; 2015.

[43] Beecham J. Collecting and estimating costs. In: Knapp M, editor. The economic evaluation of mental health care. 1st ed. Aldershot: Arena; 1995. p. 61–82.

[44] Haddock G, McCarron J, Tarrier N, Faragher EB (1999). Scales to measure dimensions of hallucinations and delusions: the Psychotic Symptom Rating Scales (PSYRATS). Psychol Med.

[45] Andreasen NC (1984). The Scale of the Assessment of Positive Symptoms (SAPS).

[46] Wessely S, Buchanan A, Reed A, Cutting J, Everitt B, Garety P (1993). Acting on delusions. I: Prevalence. Br J Psychiatry.

[47] Freeman D, Garety PA, Fowler D, Kuipers E, Bebbington PE, Dunn G (2004). Why do people with delusions fail to choose more realistic explanations for their experiences? An empirical investigation. J Consult Clin Psych.

[48] Garety P, Freeman D, Jolley S, Dunn G, Bebbington PE, Fowler DG (2005). Reasoning, emotions, and delusional conviction in psychosis. J Abnorm Psychol.

[49] Tennant R, Hiller L, Fishwick R, Platt S, Joseph S, Weich S, et al. The Warwick-Edinburgh Mental Well-being Scale (WEMWBS): development and UK validation. Health Qual Life Outcomes. 2007;5:63. 10.1186/1477-7525-5-63 PMC2222612 18042300

[50] Priebe S, Huxley P, Knight S, Evans S (1999). Application and results of the Manchester Short Assessment of Quality of Life (MANSA). Int J Soc Psychiatr.

[51] Fowler D, Freeman D, Smith B, Kuipers E, Bebbington P, Bashforth H (2006). The Brief Core Schema Scales (BCSS): psychometric properties and associations with paranoia and grandiosity in non-clinical and psychosis samples. Psychol Med.

[52] Meyer TJ, Miller ML, Metzger RL, Borkovec TD (1990). Development and validation of the Penn State Worry Questionnaire. Behav Res Ther.

[53] Lezak MDHDB, Loring DW (2004). Neuropsychological assessment.

[54] Wechsler DW (1997). Adult Intelligence Scale – Third Edition (WAIS III).

[55] Kirkpatrick B, Strauss GP, Linh N, Fischer BA, Daniel DG, Cienfuegos A (2011). The Brief Negative Symptom Scale: psychometric properties. Schizophrenia Bull.

[56] Hardy A. Thinking about Paranoia Scale. in prep.

[57] Marcus E, Garety P, Weinman J, Emsley R, Dunn G, Bebbington P (2014). A pilot validation of a modified Illness Perceptions Questionnaire designed to predict response to cognitive therapy for psychosis. J Behav Ther Exp Psy.

[58] Hooley JM, Teasdale JD (1989). Predictors of relapse in unipolar depressives—expressed emotion, marital distress, and perceived criticism. J Abnorm Psychol.

[59] Linden M (2013). How to define, find and classify side effects in psychotherapy: from unwanted events to adverse treatment reactions. Clin Psychol Psychother.

[60] Little RJA, Rubin DB. Statistical analysis with missing data. 2nd ed. Hoboken: Wiley; 2002. xv, 381.

[61] Dunn G, Maracy M, Tomenson B (2005). Estimating treatment effects from randomized clinical trials with noncompliance and loss to follow-up: the role of instrumental variable methods. Stat Methods Med Res.

[62] Dunn G, Emsley R, Liu HH, Landau S, Green J, White I (2015). Evaluation and validation of social and psychological markers in randomised trials of complex interventions in mental health: a methodological research programme. Health Technol Assess.

[63] Emsley R, Dunn G, White I (2010). Mediation and moderation of treatment effects in randomised controlled trials of complex interventions. Stat Methods Med Res.

[64] Cheong J, MacKinnon DP, Khoo ST (2003). Investigation of mediational processes using parallel process latent growth curve modeling. Struct Equ Modeling.

[65] MacKinnon DP (2008). Statistical mediation analysis.

[66] Muthén LK, Muthén BO (2016). Mplus user’s guide.

[67] World Medical Association. Declaration of Helsinki Ethical Principles for Medical Research Involving Human Subjects. JAMA. 2013;310(20):2191–4. 10.1001/jama.2013.281053 24141714

[68] Medical Research Council Guidelines for Good Clinical Practice. Available from: http://www.ich.org/products/guidelines/efficacy/article/efficacy-guidelines.html. Accessed 30 Oct 2017.

[69] Department of Health (2005). Mental Capacity Act.

[70] Freeman D, Garety P (2014). Advances in understanding and treating persecutory delusions: a review. Soc Psychiatry Psychiatr Epidemiol.

[71] Reininghaus U, Depp CA, Myin-Germeys I (2016). Ecological interventionist causal models in psychosis: targeting psychological mechanisms in daily life. Schizophr Bull.

